# The Genetic Link Between Primary Immune Thrombocytopenia and Depression/Anxiety Disorders: A Two‐Sample Mendelian Randomization Study

**DOI:** 10.1002/jcla.70176

**Published:** 2026-02-11

**Authors:** Le Jiang, Ya‐jing Zhao, Shou‐qing Han, Zhen‐yu Yan, Xin‐guang Liu

**Affiliations:** ^1^ Department of Hematology Qilu Hospital of Shandong University, Shandong University Jinan China; ^2^ Department of Hematology North China University of Science and Technology Affiliated Hospital Tangshan China

**Keywords:** anxiety, causality, depression, ITP, Mendelian randomization

## Abstract

**Background:**

Primary immune thrombocytopenia (ITP) is an autoimmune bleeding disorder with complex immunopathogenesis. Its major symptoms, such as bleeding tendency and fatigue, may predispose patients to depression and anxiety. Although psychiatric comorbidities are increasingly recognized in ITP management, whether ITP causally contributes to these conditions remains unclear.

**Methods:**

We conducted a two‐sample Mendelian randomization (MR) study to investigate the potential genetically causal links between ITP and depression/anxiety. To ensure robustness, complementary MR approaches were performed, including pleiotropy‐robust methods (MR‐Corr and MRMix), multivariable MR adjusting for inflammatory biomarkers (C‐reactive protein and interleukin‐6), the robust adjusted profile score (RAPS) model, reverse MR, and colocalization analysis.

**Results:**

MR analysis revealed a positive causal effect of ITP on depression (OR = 1.007, 95% CI: 1.001–1.013; *p* = 0.014), whereas no genetic predisposition of ITP on anxiety was observed. Multivariable and pleiotropy‐robust sensitivity analyses supported the stability and consistency of the ITP‐depression association, indicating that the result was unlikely driven by pleiotropy, instrument weakness, or inflammatory confounding.

**Conclusion:**

These findings provided novel genetic evidence supporting ITP‐associated mental health issues and highlighted the importance of integrated psycho‐hematological interventions for ITP in clinical practice. Future research was needed to elucidate the biological underpinning between ITP and psychiatric disorders, which might provide more options for ITP patients to improve life quality.

## Graphical abstract

A two‐sample Mendelian randomization study revealed a positive genetic association between primary immune thrombocytopenia (ITP) and major depressive disorder (MDD), but not with anxiety. Results remained consistent across sensitivity and multivariable analyses, indicating that ITP may causally increase susceptibility to depression.

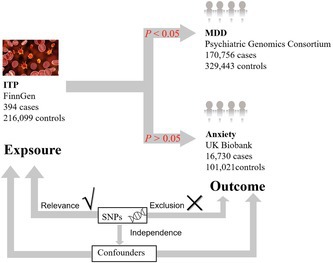

## 1 Introduction

Primary immune thrombocytopenia (ITP) is an acquired autoimmune hemorrhagic disorder characterized by isolated thrombocytopenia with heterogeneous immunopathogenesis. The pathophysiology involves immune‐mediated excessive platelet destruction in the mononuclear‐phagocyte system and compromised megakaryocyte maturation, collectively contributing to thrombocytopenia [1]. Frequent and severe bleeding, coupled with debilitating fatigue, can lead to the onset of depression and anxiety, significantly compromising the health‐related quality of life in ITP patients [2]. Psychiatric disorders have attracted increasing attention as they profoundly impair patients' daily functioning and may even lead to unexpected mortality if not effectively managed [3].

Accumulating epidemiological studies have revealed a significantly increased prevalence of depression and anxiety in ITP populations compared to the age‐matched controls. A cross‐sectional study of the pediatric age group in Turkey showed that approximately half of the patients with ITP scored higher both on the anxiety and depression scales compared to the control populations [4]. Consistently, another cohort study revealed that the prevalence of depression in adult patients with ITP was 25% in the United Kingdom and 16% in the United States, corresponding to 8.3‐fold and 8.0‐fold increases, respectively, compared to the matched healthy populations [5]. Additionally, adult patients with ITP were at a higher risk of experiencing mental health events and greater utilization of psychotropic medications compared to the general population, with an obvious positive correlation between ITP diagnosis and psychiatric morbidity, indicating an intrinsic link in the pathogenesis of ITP and psychiatric disorders [6].

To investigate the potential genetic correlations between ITP and depression/anxiety, we employed the two‐sample Mendelian randomization (MR) framework. This causal inference approach leverages genetic variants as instrumental variables, capitalizing on the random allocation of genotypes during meiosis to minimize confounding factors in exposure‐outcome association analyses. Given the complex immune and psychiatric pathophysiology and the potential for horizontal pleiotropy, we further applied several complementary MR approaches to strengthen causal inference. Specifically, we incorporated pleiotropy‐robust methods (MR‐Corr and MRMix), multivariable MR (MVMR) analyses adjusting for systemic inflammatory biomarkers (CRP and IL‐6), and reverse MR to assess the alternative causal direction. These additional analyses were designed to mitigate bias arising from correlated pleiotropy, weak instruments, and inflammatory confounding, thereby providing a more rigorous and comprehensive evaluation of the potential causal relationship between ITP and mood disorders. Unraveling the genetic correlations between ITP and neuropsychiatric comorbidities may offer valuable insights into their shared pathophysiology and facilitate the development of targeted therapeutic strategies for these clinically challenging concurrent conditions.

## 2 Materials and Methods

### 2.1 Mendelian Randomization Study

**FIGURE 1:**
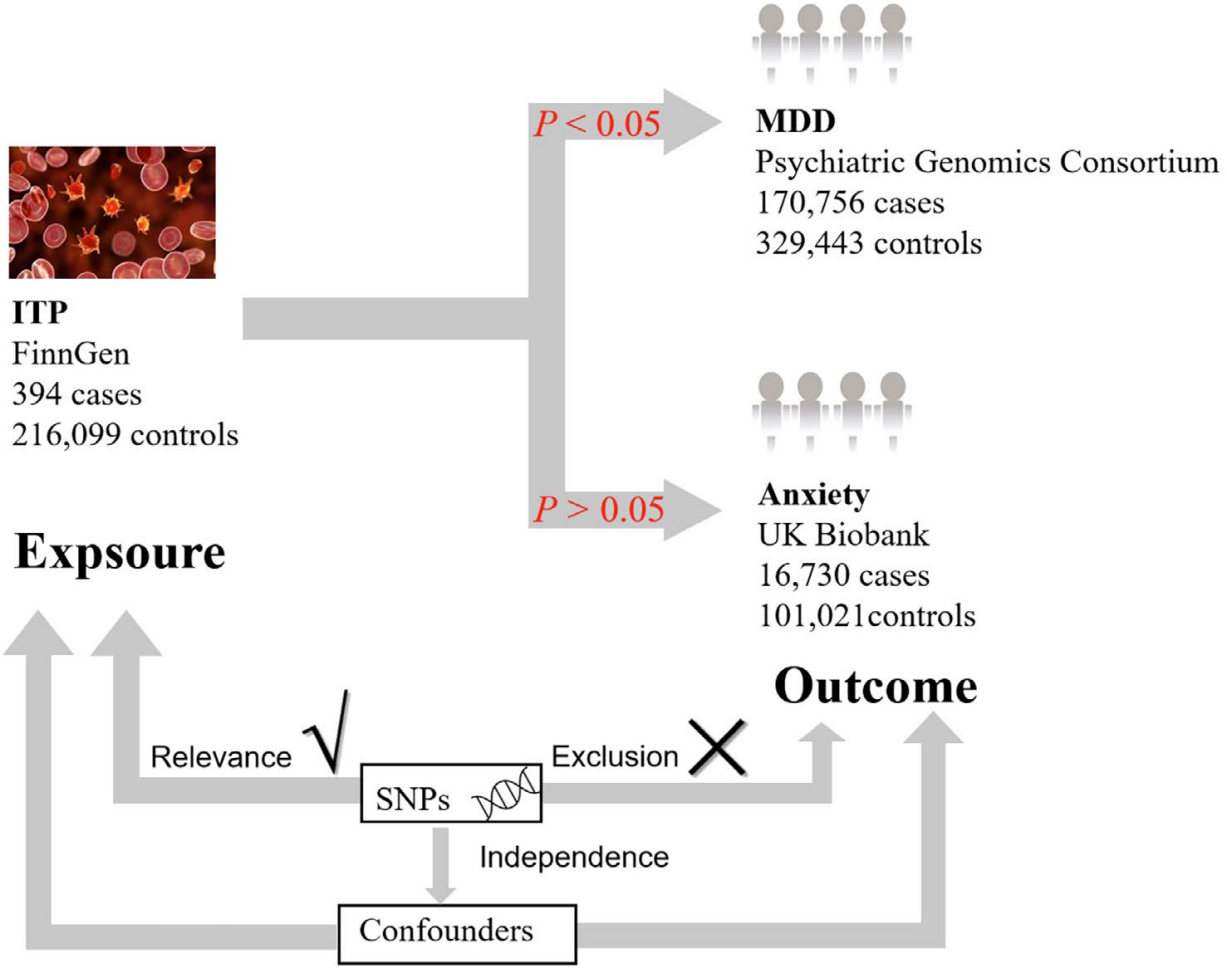
Overview of the Mendelian randomization (MR) study design. The design of the two‐sample MR study was displayed with key components including genetic datasets and methodological considerations. Data sources comprise the large‐scale cohorts: FinnGen (ITP: 394 cases, 216,099 controls), the Psychiatric Genomics Consortium (MDD: 170,756 cases, 329,443 controls), and UK Biobank (Anxiety: 16,730 cases, 101,021 controls). Methodological rigor was ensured through SNP selection, independence testing, exclusion of confounding variants, and adjustment for potential confounders. GWAS, genome‐wide association study; ITP, primary immune thrombocytopenia; MDD, major depressive disorder; SNP, single nucleotide polymorphism.

This two‐sample Mendelian randomization (MR) analysis was conducted to evaluate potential causal relationships between ITP and depression/anxiety. The key analytical components were schematized in Figure 1. The validity of MR‐derived causal inferences required the satisfaction of three fundamental assumptions: (i) Relevance assumption: genetic instruments must demonstrate genome‐wide significant association with ITP status; (ii) Independence assumption: instrumental variables should be independent of known or putative confounders affecting the ITP‐depression/anxiety relationship; (iii) Exclusion restriction: genetic instruments must influence the outcome exclusively through the exposure pathway.

### 2.2 Data Sources

Genetic instruments for ITP exposure were obtained from the FinnGen consortium, a collaborative research project comprising 394 clinically verified ITP cases and 216,099 population‐matched controls [7]. Major depressive disorder (MDD) outcome data originated from the Psychiatric Genomics Consortium's (PGC) genome‐wide meta‐analysis dataset, which integrates 170,756 MDD cases and 329,443 controls collected across 33 population‐based studies [8]. Anxiety disorder phenotypes were extracted from the UK Biobank GWAS (data field 20544, instance 15), comprising 16,730 ICD‐10 diagnosed cases and 101,021 controls (MRC‐IEU GWAS ID: ukb‐d‐20544_15). For multivariable Mendelian randomization (MVMR) analyses, summary statistics for systemic inflammatory biomarkers were obtained from large‐scale GWAS datasets. C‐reactive protein (CRP) levels were obtained from the Within‐Family GWAS Consortium study (IEU OpenGWAS ID: ieu‐b‐4764), which included 61,308 participants of European ancestry. Interleukin‐6 (IL‐6) concentrations were extracted from the Proteome GWAS dataset (GWAS ID: prot‐a‐1539), comprising 3301 European individuals.

### 2.3 Selection of Instrumental Variables

We selected single nucleotide polymorphisms (SNPs) as instrumental variables (IVs). Given the limited number of genome‐wide significant variants (*p* < 5 × 10^−8^) available for ITP in the discovery cohort, variants meeting genome‐wide suggestive significance (*p* < 1 × 10^−5^) were prioritized to optimize instrument strength. To ensure genetic independence, linkage disequilibrium (LD) clumping (*r* ^2^ = 0.001, 10‐Mb window) was performed using European ancestry data from Phase 3 of the 1000 Genomes Project as the reference panel. [9] The strength of each IV was quantified via the *F* statistic, calculated as the square of the SNP's exposure association (*β* ^2^) divided by the square of its standard error (SE^2^). Variants with *F* < 10, indicative of potential weak instrument bias, were excluded in accordance with MR sensitivity analysis recommendations [10]. To quantify the strength of the genetic instruments and assess potential weak instrument bias, we calculated the proportion of variance explained (*R* ^*2*^) by all SNPs in the exposure (ITP) using the formula:

$$R^{2}=\frac{2\beta^{2}\mathrm{MAF}\left(1-\mathrm{MAF}\right)}{2\beta^{2}\mathrm{MAF}\left(1-\mathrm{MAF}\right)+2\text{NMAF}\left(1-\mathrm{MAF}\right)SE^{2}}$$

where *β* is the effect size per allele, MAF is the minor allele frequency, SE is the standard error of *β*, and N is the total sample size of the ITP GWAS (394 cases and 216,099 controls for ITP, yielding N = 216,493) [11]. To assess potential violations of the instrumental variable independence assumption, we systematically queried each ITP‐associated SNP using the OpenGWAS database to identify associations with known confounders, including BMI, smoking, and autoimmune diseases (e.g., systemic lupus erythematosus) [12]. SNPs showing genome‐wide (*p* < 5 × 10^−8^) or suggestive (*p* < 1 × 10^−5^) associations with any confounder were flagged for exclusion.

### 2.4 The Univariable Mendelian Randomization Analysis and Sensitivity Testing

We employed 3 different methods—including MR‐Egger, weighted median, and random‐effect inverse variance weighted (IVW)—to conduct the MR analysis [13]. When all selected SNPs satisfied the IV assumptions, the IVW method was used as the primary analytical approach because it provides the most statistically efficient causal estimates under valid instruments [14]. MR‐Egger regression, which can detect and correct for directional pleiotropy [15], and the weighted median method, which derives causal estimates from the median of variant‐specific associations [16], were employed as complementary approaches, acknowledging their reduced statistical efficiency compared to IVW. To ensure analytical robustness, we conducted comprehensive sensitivity analyses using established MR methodologies. The MR‐Pleiotropy RESidual Sum and Outlier (MR‐PRESSO) method was employed to assess horizontal pleiotropy through 3 distinct components: (1) global test for overall pleiotropy, (2) outlier detection analysis, and (3) distortion test to evaluate effect estimate adjustments [17]. We performed Cochran's *Q* test to evaluate instrument heterogeneity, where a nonsignificant result (*p* > 0.05) suggested consistency across genetic variants in their outcome effects [18]. The leave‐one‐out sensitivity analysis was performed by iteratively excluding individual SNPs and repeating the IVW analysis. Substantial alterations in effect estimates following specific SNP exclusion would suggest potential influential variants or bias introduction [18]. All effect estimates were expressed as odds ratios (ORs) with 95% confidence intervals (CIs), appropriate for our dichotomous outcome measures (depression and anxiety diagnoses).

### 2.5 Sensitivity Analyses Using Pleiotropy‐Robust MR Approaches (MR‐Corr and MRMix)

To further verify the robustness of causal inference and address potential pleiotropy, we implemented two contemporary methods: MR‐Corr and MRMix. MR‐Corr models correlated pleiotropy by incorporating the covariance structure among genetic instruments, thereby mitigating bias arising from correlated horizontal pleiotropy [19]. An LD matrix was constructed from the *1000 Genomes project* European reference panel to account for linkage disequilibrium among SNPs [9]. MRMix employs a mixture‐model framework that distinguishes valid from invalid instruments and provides consistent causal estimates even when a subset of variants violates MR assumptions. The causal parameter was estimated using a grid search over θ (−2 to 2, step = 0.01) with profile likelihood optimization [20]. Both analyses were performed in R using the *MR.Corr2* and *MRMix* packages, with harmonized summary statistics from the same exposure‐outcome pairs as in the primary MR analyses. Results were reported as ORs with 95% CI derived from the estimated *β* coefficients.

### 2.6 Multivariable Mendelian Randomization

To investigate whether systemic inflammatory biomarkers might confound the causal pathway between ITP and MDD, we conducted multivariable MR (MVMR) including CRP and IL‐6 as additional exposures [21]. For each exposure we first extracted genome‐wide significant SNPs (primary threshold *p* < 5 × 10^−8^) and, where instruments were sparse, re‐extraction was performed using a relaxed threshold (*p* < 1 × 10^−5^) to assemble a sufficient instrument set. SNPs across exposures were combined and LD‐clumped (*r* ^2^ = 0.001, 10‐Mb window) to obtain an approximately independent union set. All instruments were harmonized across datasets. Causal estimates were derived using the multivariable IVW method as the primary model. Conditional *F*‐statistics were calculated to evaluate the strength of each exposure‐specific instrument set, where values above 10 indicated adequate instrument strength [22]. The estimated causal effects were expressed as beta coefficients (*β*) and corresponding standard errors (SE), with statistical significance assessed using *p* values. When the conditional *F*‐statistic was below 10, we applied the robust adjusted profile score (RAPS) method to mitigate weak instrument bias. RAPS provides consistent estimates and valid inference under weak instrument conditions by using robust regression with a Huber loss function to downweight outliers, outperforming IVW methods [23].

### 2.7 Reverse Mendelian Randomization Analysis

We also performed a reverse two‐sample MR analysis using independent SNPs associated with MDD or anxiety as the exposure instruments and ITP as the outcome to examine the potential reverse causation from MDD to ITP. The same analytical framework as the forward MR (IVW as the main method, complemented by MR‐Egger, weighted median, and MR‐PRESSO) was applied.

### 2.8 Colocalization Analysis

To determine whether the observed causal association between ITP and MDD was attributable to shared genetic variants within the same genomic regions, we performed colocalization analysis. For each of the lead SNPs identified in the primary MR analysis, we extracted summary‐level association statistics for all variants within a ± 500 kb window from the corresponding GWAS datasets of ITP and MDD from the IEU OpenGWAS project as VCF files [24, 25]. The *coloc.abf* function was applied to compute the posterior probabilities for five mutually exclusive hypotheses: H0 (*PP0*): no association with either trait; H1 (*PP1*): association with ITP only; H2 (*PP2*): association with MDD only; H3 (*PP3*): both traits are associated but with distinct causal variants; H4 (*PP4*): both traits share a single causal variant [26]. Evidence for colocalization was classified as strong when *PP4* > 0.75, moderate when 0.5 ≤ *PP4* ≤ 0.75, and weak or absent when *PP4* < 0.5.

### 2.9 Statistical Analysis and Implementation

All analyses were performed using R statistical software (v4.4.1; RStudio 2024.09.0+375). The following packages were used: *TwoSampleMR* for the main MR analyses, *MRPRESSO* for pleiotropy correction, *MRMix* for mixture‐model estimation, and *MRCorr2* for correlation‐adjusted MR. Multivariable MR analyses were implemented using the *MVMR* and *TwoSampleMR* frameworks, and colocalization was performed using the *coloc* package.

## 3 Results

### 3.1 Selection and Strength of Instrumental Variables

Using the predefined selection criteria, 23 ITP‐associated SNPs were identified, all with *F*‐statistics exceeding the conventional threshold of 10 (minimum *F* = 19.54). Collectively, these instruments explained approximately 0.23% of the variance in ITP risk (total *R* ^2^ = 0.00232), with a mean *F*‐statistic of 21.82 (range: 19.54–28.02). These results indicated that the selected instruments possessed adequate strength, thereby minimizing the potential for weak instrument bias in the Mendelian randomization analysis. The detailed characteristics and strength metrics (*R* ^2^ and *F*‐statistics) for all SNPs were presented in Table S1. No genome‐wide significant (*p* < 5 × 10^−8^) or suggestive (*p* < 1 × 10^−5^) associations were identified between the instrumental SNPs for ITP and known confounding traits such as BMI, smoking, autoimmunity, or CRP. These findings supported the validity of the independence assumption for the selected instruments.

### 3.2 Primary MR Estimates Revealed a Genetic Predisposition of ITP on MDD

Exposure‐outcome datasets were harmonized using the *TwoSampleMR* package, aligning SNP characteristics (including alleles, effect sizes, and frequencies) while flagging palindromic variants with ambiguous strand orientation. Following this procedure, a total of 17 SNPs (excluding 2 palindromic SNPs: rs117283193, rs117924460) were retained to investigate the causal relationship of ITP on MDD, and 20 SNPs (excluding three palindromic SNPs: rs117283193, rs117924460, rs6773857) were retained to explore the causal linkage of ITP on anxiety. Details of the selected SNPs, including effect alleles, beta coefficients, and *p* values, were documented in Tables S2 and S3.

**FIGURE 2:**
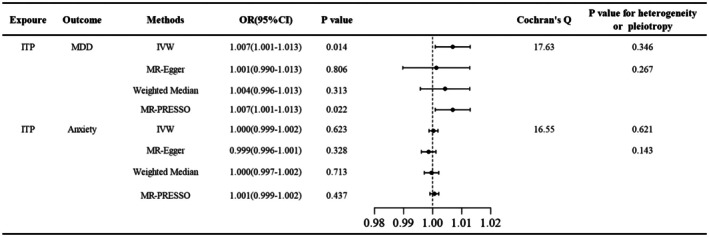
The two‐sample MR estimates of ITP on MDD/anxiety. The IVW and MR‐PRESSO method showed that ITP was associated with an increased risk of MDD (IVW: *p* = 0.014; MP‐PRESSO: *p* = 0.022). No significant association was observed between ITP and anxiety. Cochran's *Q* test demonstrated homogeneity across IVs for both MDD and anxiety. 95% CI, 95% confidence interval; IVs, instrumental variances; IVW, inverse variance weighted; MDD, major depressive disorder; MR‐PRESSO, MR‐Pleiotropy RESidual Sum and Outlier; OR, odds ratio.

**FIGURE 3:**
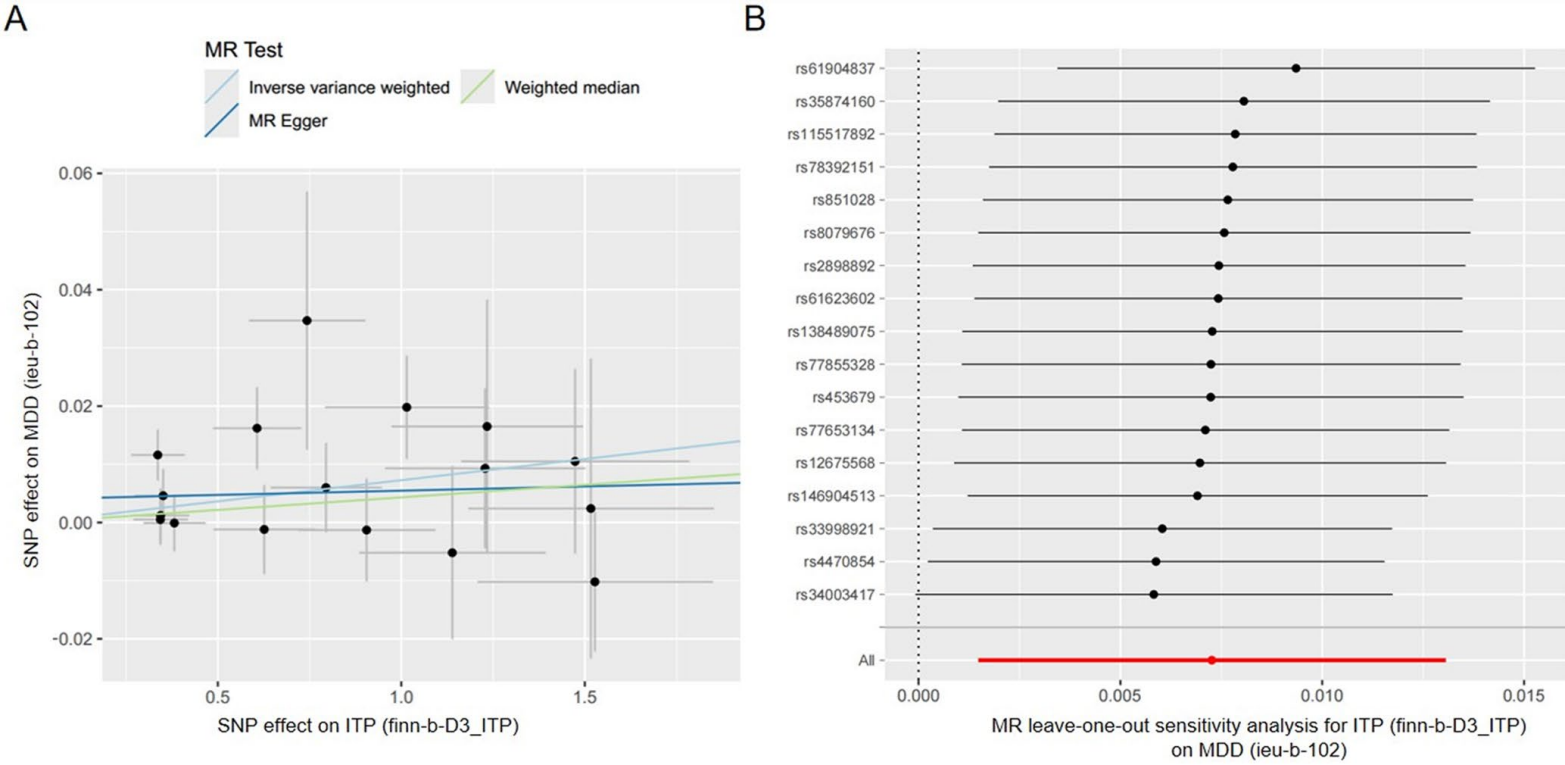
The genetic correlation between ITP and MDD through MR analysis. The exposure‐outcome associations between ITP and MDD were depicted through scatter plots of SNP, where regression slopes represented causal effect estimates derived from different MR methods including random‐effect IVW, MR‐Egger, and weighted median. (B) The leave‐one‐out sensitivity analysis of the effect of ITP on MDD showed that the exclusion of any individual SNP had a negligible impact on the overall result. IVW, inverse variance weighted; MDD, major depressive disorder; SNP, single nucleotide polymorphism.

The MR‐Egger intercept test revealed no evidence of directional pleiotropy for either outcome (MDD: *p* = 0.267; anxiety: *p* = 0.143; Figure 2). Cochran's *Q* test demonstrated homogeneity across IVs for both MDD (*Q* = 17.63, *p* = 0.346) and anxiety (*Q* = 16.55, *p* = 0.621). IVW analysis identified a genetically predicted increased MDD risk in ITP population (OR = 1.007, 95% CI: 1.001–1.013, *p* = 0.014). Although MR‐Egger (OR = 1.001, *p* = 0.806) and weighted median (OR = 1.004, *p* = 0.313) estimates did not reach statistical significance, their effect directions aligned with the IVW results (Figures 2 and 3A). MR‐PRESSO analysis, as presented in Figure 2, corroborated the IVW findings, showing a consistent positive correlation of ITP on MDD (OR = 1.007, 95% CI: 1.001–1.013, *p* = 0.022). The leave‐one‐out sensitivity analysis (Figure 3B) demonstrated that the exclusion of any individual SNP had negligible impact on the overall results, confirming the robustness of the genetic association of ITP on MDD.

By contrast, IVW analysis revealed no statistically significant genetic predisposition of ITP on anxiety risk (OR = 1.000, 95% CI: 0.999–1.002, *p* = 0.623, Figure 2). The null association was visually corroborated by regression lines demonstrating slopes approximating zero (Figure S1A). The leave‐one‐out sensitivity analysis further confirmed the robustness of the null finding, with confidence intervals persistently overlapping zero, indicating that the null association was not driven by any single influential SNP (Figure S1B).

### 3.3 Robustness Analyses Using Pleiotropy‐Robust MR Methods (MR‐Corr and MRMix)

To further evaluate the robustness of the causal inference against correlated and unbalanced horizontal pleiotropy, pleiotropy‐robust Mendelian randomization models were applied, including MR‐Corr and MRMix analyses (Table S4). Both approaches yielded effect estimates close to the null (MR‐Corr: *β* = −4.0 × 10^−4^, SE = 3.61 × 10^−4^, OR = 1.000 [95% CI 0.999–1.001], *p* = 0.264; MRMix: *β* = 0, SE = 0.00055, OR = 1.000 [95% CI 0.999–1.001], *p* = 1). The high proportion of valid instrumental variables detected by MRMix (*π*₀ = 0.972) and the minimal pleiotropy variance (*σ* ^2^ = 1.24 × 10^−5^) indicated that the primary IVW results were unlikely to be biased by pleiotropic effects [20]. These findings collectively supported the robustness of the observed positive association between genetically predicted ITP and MDD.

### 3.4 Multivariable MR Adjusting for CRP and IL‐6 Confirmed the Causal Relationship Between ITP and MDD

**FIGURE 4:**
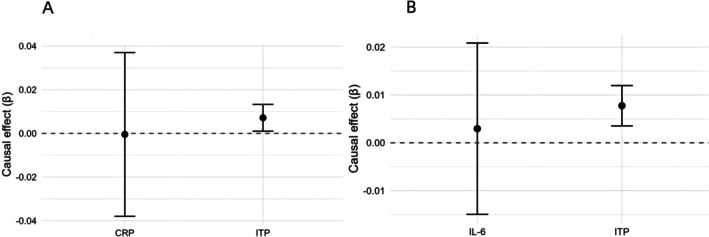
Multivariable Mendelian randomization analyses of ITP and inflammatory biomarkers on MDD. Forest plots showing causal estimates (*β*) and 95% CI from MVMR analyses of ITP and inflammation biomarkers on MDD. (A) ITP was associated with MDD when adjusted for CRP (*p* = 0.027); CRP showed no effect on MDD (*p* = 0.980). (B) ITP remained associated with MDD after adjusted for IL‐6 (*p* = 0.013), while IL‐6 had no effect on MDD (*p* = 0.815). 95% CI, 95% confidence interval; CRP, C‐reactive protein; IL‐6, Interleukin‐6; ITP, primary immune thrombocytopenia; MDD, major depressive disorder; MVMR, multivariable Mendelian randomization; *β*, beta coefficient (causal effect estimate).

To determine whether the causal effect of ITP on MDD was mediated by systemic inflammation, we conducted MVMR analyses adjusting for CRP and IL‐6. After accounting for CRP, the association between genetically predicted ITP and MDD remained statistically significant (*p* = 0.027), whereas CRP showed no independent effect on MDD (*p* = 0.980). Similarly, when adjusting for IL‐6, the effect of ITP on MDD persisted (*p* = 0.013), while IL‐6 was not associated with MDD (*p* = 0.815). Given that the *F*‐statistic for ITP in the ITP + CRP model (*F* = 9.12) was slightly below the conventional threshold of 10, suggesting potential weak instrument bias, we performed additional robust adjusted profile score (RAPS) analysis to validate our findings. The RAPS analysis confirmed the robustness of our results, showing consistent effect estimates for ITP on MDD (*β* = 0.007, SE = 0.003, *p* = 0.038) and null effects for CRP (*β* = −0.002, SE = 0.019, *p* = 0.906). The remarkable consistency between MVMR‐IVW and RAPS estimates reinforced the reliability of our primary findings despite borderline instrument strength. These results suggested that the observed causal relationship between ITP and MDD was unlikely to be confounded by systemic inflammation markers such as CRP or IL‐6 (Table 1 and Figure 4).

**TABLE 1:** Multivariable Mendelian randomization (MVMR) estimates for the effect of ITP, CRP, and IL‐6 on MDD.

Exposure model	Exposure	*β*	SE	*p*	Conditional *F*
ITP + CRP → MDD	ITP	0.007	0.003	0.027	9.12
	CRP	−0.001	0.019	0.980	51.96
ITP + IL‐6 → MDD	ITP	0.008	0.003	0.013	11.42
	IL‐6	−0.002	0.009	0.815	12.07

Abbreviations: Conditional *F*, conditional *F*‐statistic for instrument strength in the multivariable model; SE, standard error; *β*, effect estimate.

### 3.5 Reverse MR Analysis Showed no Causal Effect of MDD on ITP

A total of 213 independent SNPs associated with MDD at a suggestive genome‐wide significance threshold (*p* < 1 × 10^−5^) were initially extracted as instrumental variables. After excluding palindromic variants, and two outliers (rs7098208; rs75520450) identified by the MR‐PRESSO global test, 183 SNPs were retained for the reverse MR analysis. The inverse variance weighted (IVW) method indicated no significant genetic causal effect of MDD on ITP (OR = 1.036, 95% CI: 0.632–1.701, *p* = 0.888). Both MR‐Egger (OR = 0.317, 95% CI: 0.047–2.135, *p* = 0.239) and weighted median (OR = 1.083, 95% CI: 0.538–2.181, *p* = 0.824) approaches yielded consistent null results with concordant effect directions. The MR‐Egger intercept test indicated no evidence of directional pleiotropy (*p* = 0.209), and Cochran's *Q* statistics showed no significant heterogeneity across the instruments (IVW: *Q* = 207.37, *p* = 0.096; MR‐Egger: *Q* = 205.56, *p* = 0.102). Collectively, these findings suggested that there was no genetic evidence supporting a causal effect of MDD on ITP. All detailed results were summarized in Table 2 and Table S5. Reverse MR for anxiety‐ITP was also attempted; however, the obtained odds ratios were highly imprecise with extremely wide 95% confidence intervals, indicating instability due to limited instrument strength [27]. Therefore, these results were not presented in the main text but are available upon request.

**TABLE 2:** Mendelian randomization estimates for the causal effect of MDD on ITP (reverse‐direction analysis).

MR method	*β*	SE	OR (95% CI)	*p*	Cochran's *Q* (*p*)	MR‐Egger intercept (*p*)
IVW	0.036	0.253	1.036 (0.632–1.701)	0.888	207.365 (0.096)	—
MR‐Egger	−1.150	0.974	0.317 (0.030–1.638)	0.239	205.560 (0.102)	0.033 (0.209)
Weighted median	0.079	0.357	1.083 (0.538–2.181)	0.824	—	—
MR‐PRESSO (outlier‐corrected)	0.0576	0.242	1.059 (0.659–1.702)	0.812	—	—

Abbreviations: 95% CI, 95% confidence interval; IVW, inverse variance weighted; MR‐Egger intercept tests pleiotropy; MR‐PRESSO, MR pleiotropy residual sum and outlier. Cochran's *Q* tests heterogeneity; OR, odds ratio; SE, standard error; *β*, causal effect estimate.

### 3.6 Colocalization Analysis of the Lead SNPs

To investigate whether the observed causal association between ITP and MDD was driven by the same underlying genetic variants, we conducted colocalization analysis using a ±500 kb window around each of the 17 lead SNPs. Across all loci, posterior probabilities for a shared causal variant (*PP4*) were consistently low (all *PP4* < 0.1, mean *PP4* ≈ 0.01, Table S6). In contrast, most regions showed high *PP0* (no association in either trait) or *PP1* (association with ITP only), while a few regions exhibited moderate *PP2* or *PP3*, indicating independent signals. Notably, none of the loci reached the commonly accepted threshold for strong colocalization evidence (*F* ≥ 0.8) (Figure S2). These results suggested that the association between ITP and MDD detected in the MR analysis was unlikely to be driven by the same causal variant at specific loci, but might instead reflect genome‐wide polygenic correlation or unmeasured confounding.

## 4 Discussion

Accumulating evidence has demonstrated that patients with ITP have considerably elevated prevalence of depression and anxiety compared to control populations [5, 6]. Bleeding, the major clinical manifestation of ITP, ranges from minor mucocutaneous bleeding to life‐threatening intracranial hemorrhage, with the severity of bleeding often discordant with platelet counts. The unpredictable nature of bleeding risk in daily life largely contributes to the health‐related anxiety in ITP patients [28, 29]. Additionally, fatigue, reported in 22%–45% of ITP patients, exacerbates physical disability and psychological distress, further compounding the disease burden [30, 31, 32]. The ITP World Impact Survey study revealed nearly half of the ITP patients experienced clinically significant psychological burden, predominantly driven by: (1) anxiety over disease monitoring parameters, (2) concerns about disease progression, and (3) frustration with persistent symptoms despite therapy [33]. Notably, a recent NHANES‐based analysis found no independent association between ITP medications and depression risk, suggesting that psychological comorbidities in ITP patients might arise predominantly from disease‐related stressors rather than pharmacological interventions [34]. These findings underscore the urgent need to incorporate standardized mental health screening and evidence‐based psychosocial interventions in ITP management protocols. Current knowledge gaps necessitate rigorous investigations of targeted biopsychosocial interventions for ITP‐related neuropsychiatric comorbidities. Nevertheless, the pathophysiological mechanisms linking immune dysregulation in ITP to neuropsychiatric manifestations remain poorly elucidated.

The present two‐sample MR study provided novel genetic evidence by examining the putative causal relationship of ITP on depression/anxiety. By leveraging the random assortment of genetic variants at conception, the MR could minimize confounding bias and reverse causation inherent in observational studies [13]. The IVW method revealed a positive association of ITP on depression in the genetic settings, which was confirmed by MR‐PRESSO analysis, indicating the genetic predisposition to psychiatric disorders in ITP. These findings aligned with the previous observational studies reporting an increased incidence of psychiatric disorders in ITP patients [4, 5, 6]. Robust sensitivity analyses further strengthened the validity of the causal inference.

In addition to the primary MR findings, several complementary analyses were performed to assess the robustness and potential mechanisms underlying the observed associations. Additional pleiotropy‐robust analyses (MR‐Corr and MRMix) yielded consistent null estimates after accounting for correlated and unbalanced pleiotropy, indicating that the observed genetic association between ITP and MDD is unlikely to be an artifact of pleiotropic bias [19, 20]. The minimal pleiotropy variance and high proportion of valid instruments (π₀ ≈ 0.97) further supported the stability of the causal estimate across complementary MR frameworks. It should also be noted that both MR‐Corr and MRMix are conservative approaches, and their effect estimates may be attenuated when the number of available instruments is modest, as in the present study [19, 35]. Therefore, these results should be interpreted as supporting the validity and robustness of the primary causal estimate rather than indicating the absence of a causal effect. Moreover, multivariable MR adjusting for systemic inflammatory biomarkers (CRP and IL‐6) further demonstrated that the causal effect of ITP on MDD was not simply mediated by systemic inflammation [21]. Although the *F*‐statistic for ITP in the multivariable model adjusting for CRP was slightly below the conventional threshold, RAPS analysis produced concordant results with null effects for CRP, implying minimal influence from weak instruments. The RAPS approach, which downweights influential variants and corrects for weak instrument bias and idiosyncratic pleiotropy, has been used to provide robust inference even under imperfect instrument strength [23, 36]. The reverse MR analysis provided no evidence supporting a causal effect of MDD on ITP, confirming the one‐sided genetic predisposition of ITP on MDD. Finally, colocalization analysis revealed no shared causal variants (all *PP*₄ < 0.1), consistent with a polygenic correlation rather than a single shared locus [26]. Instead, consistent with methodological insights from recent studies, such a pattern typically indicated that the genetic relationship between ITP and MDD was driven by broad polygenic correlation rather than a single shared causal variant; alternatively, it may reflect distinct but biologically related variants that influence immune and psychiatric pathways in parallel. [37] Collectively, these findings reinforced the robustness, specificity, and directionality of the causal inference linking ITP to depression.

Beyond the psychosocial stressors inherent to chronic illness, our findings raised the possibility of shared biological pathways linking ITP and depression. The observed association likely reflects complex immune‐neural interactions. Although our multivariable MR results indicated that systemic inflammatory markers, including IL‐6 and CRP, did not fully account for the observed genetic association between ITP and MDD, this association may involve other immune‐neural mechanisms. These include localized neuroinflammation driven by microglial activation, platelet‐derived serotonin or other mediators affecting monoaminergic signaling, and immune‐driven alterations in the tryptophan‐kynurenine pathway that yield neurotoxic metabolites [38, 39, 40]. Moreover, shared genetic and transcriptomic pathways involving cytokine signaling, hypothalamic–pituitary–adrenal (HPA) axis regulation, and synaptic plasticity may underlie the observed polygenic overlap between autoimmune and psychiatric phenotypes [41, 42, 43]. Collectively, these findings supported a multifactorial immune‐neural interface linking ITP and depression and highlighted the need for mechanistic and longitudinal investigation to delineate its causal underpinnings.

By contract, no significant genetic correlation was found between ITP and anxiety, which might be due to the distinct biological and genetic underpinnings of anxiety and depression. However, this null association should be interpreted with caution, as anxiety phenotypes differ substantially across GWASs in case definition and severity. The anxiety GWAS used in this study was based on ICD‐10 clinical diagnoses from the UK Biobank, likely capturing more severe or treatment‐seeking cases while underrepresenting subthreshold or generalized anxiety symptoms common in the community [44]. Previous studies have demonstrated that the genetic architecture of ICD‐coded anxiety disorders only partially overlaps with questionnaire‐based anxiety phenotypes (rg ≈ 0.6–0.7) [44, 45]. Additionally, instrumental variables derived from ITP‐associated loci may have limited explanatory power when applied to outcome datasets representing narrower or clinically heterogeneous phenotypes, which could attenuate the observed causal effect. The modest number of anxiety cases (*n* = 16,730) also constrains statistical power, increasing the risk of false negatives. Future MR studies using larger meta‐analytic datasets or refined anxiety subtypes (e.g., generalized anxiety, social anxiety, panic disorder) are warranted to delineate potential subtype‐specific genetic links with ITP.

Notwithstanding these findings, several limitations of this study warrant consideration. First, given the modest discovery cohort size and the absence of genome‐wide significant SNPs for ITP (*p* < 5 × 10^−8^), a less stringent threshold (*p* < 1 × 10^−5^) was applied to retain sufficient instruments, which may introduce pleiotropic bias—a recognized limitation of MR studies. Nevertheless, all selected SNPs had *F*‐statistics above 10, supporting adequate instrument strength. Second, as the genetic datasets were derived exclusively from European populations, the generalizability of our results to other ethnic groups may be limited; replication in more diverse cohorts is warranted. Third, the limited availability of large individual‐level ITP datasets prevented the use of polygenic approaches such as MR‐polygenic risk score (MR‐PRS) and also precluded independent replication [46]. Future large‐scale studies will be essential to provide more comprehensive validation. Finally, the observed causal estimate of ITP on MDD was modest (OR = 1.007); nevertheless, it was statistically robust and consistent across multiple complementary MR sensitivity approaches (MR‐PRESSO, MR‐Corr, MRMix, MVMR, and RAPS). Such small but statistically reproducible genetic effects are typical for complex polygenic traits, where multiple variants cumulatively contribute to disease susceptibility [47, 48]. From a population perspective, even subtle shifts in genetic risk may carry meaningful public health implications given the high prevalence of depression [49]. Therefore, while the clinical effect of ITP on depression may be small at the individual level, the observed genetic link provides valuable mechanistic evidence supporting immune‐neural interactions that warrant further functional exploration [38].

In conclusion, our MR analysis provided novel evidence supporting a potential causal relationship of ITP on depression. These findings underscored the clinical imperative to integrate standardized psychological care into the multidisciplinary management of ITP patients. Regular depression screening, such as using the Patient Health Questionnaire‐9 (PHQ‐9), should be prioritized for ITP patients, with prompt referral to mental health specialists when necessary to optimize patient‐centered care [50]. Further mechanistic studies are warranted to elucidate the neuro‐immunological pathways linking platelet autoimmunity to psychiatric manifestations. Such research could reveal novel therapeutic targets bridging hematological and mental health interventions, ultimately enabling precision medicine approaches in ITP management.

## Author Contributions

L.J. performed research, analyzed data, and wrote the paper. Y.Z. and X.L. evaluated the data and corrected the paper. S.H. provided guidance on data analysis techniques. X.L. and Y.Z. designed and reviewed the work; and all authors read and edited the manuscript.

## Funding

This study was supported by the National Natural Science Foundation of China (grant no. 82170123), the Natural Science Foundation of Shandong Province (ZR2021MH132), and the Young Taishan Scholar Foundation of Shandong Province (grant/award no. tsqn202312325).

## Ethics Statement

Data analyzed in the MR analysis was derived from publicly available GWAS genetic aggregation data and therefore requires no additional ethical approval or participant consent. All original studies have been approved by the corresponding ethical review board, and the participants have signed informed consent.

## Consent

The authors have nothing to report.

## Conflicts of Interest

The authors declare no conflicts of interest.

## Supporting information


**Data S1:** jcla70176‐sup‐0001‐Supinfo.docx.

## Data Availability

The exposure data on genetic variations associated with ITP were gathered from the FinnGen consortium, a collaborative research project that unites geneticists, biologists, and medical professionals from across Finland, with the MRC‐IEU GWAS ID: finn‐b‐D3_ITP. The data of MDD were retrieved from 33 studies conducted by the Psychiatric Genomics Consortium, with the MRC‐IEU GWAS ID: ieu‐b‐102. The anxiety disorder data came from the UK Biobank (a large, population‐based cohort consisting of 503,325 individuals that were included by general practitioners of the UK National Health Service between 2006 and 2010, with the MRC‐IEU GWAS ID: ukb‐d‐20544_15). C‐reactive protein (CRP) levels were obtained from the Within‐Family GWAS Consortium study (IEU OpenGWAS ID: ieu‐b‐4764), which included 61308 participants of European ancestry. Interleukin‐6 (IL‐6) concentrations were extracted from the Proteome GWAS dataset (GWAS ID: prot‐a‐1539), comprising 3301 European individuals.
