# Supplementary material for: The Genetic Link Between Primary Immune Thrombocytopenia and Depression/Anxiety Disorders: A Two‐Sample Mendelian Randomization Study
Source: J Clin Lab Anal. 2026 Feb 11;40(6):e70176. doi: 10.1002/jcla.70176 (PMC13042916; doi:10.1002/jcla.70176)
Supplement: Supplementary file 1 — Data S1: jcla70176‐sup‐0001‐Supinfo.docx. [file JCLA-40-e70176-s001.docx]

**The genetic link between primary immune thrombocytopenia and depression/anxiety disorders: a two-sample Mendelian randomization study**

**Running title**: Genetic link between ITP & mood disorders

Le Jiang, Ya-jing Zhao, Shou-qing Han, Zhen-yu Yan, Xin-guang Liu

**Supplementary figures**

**
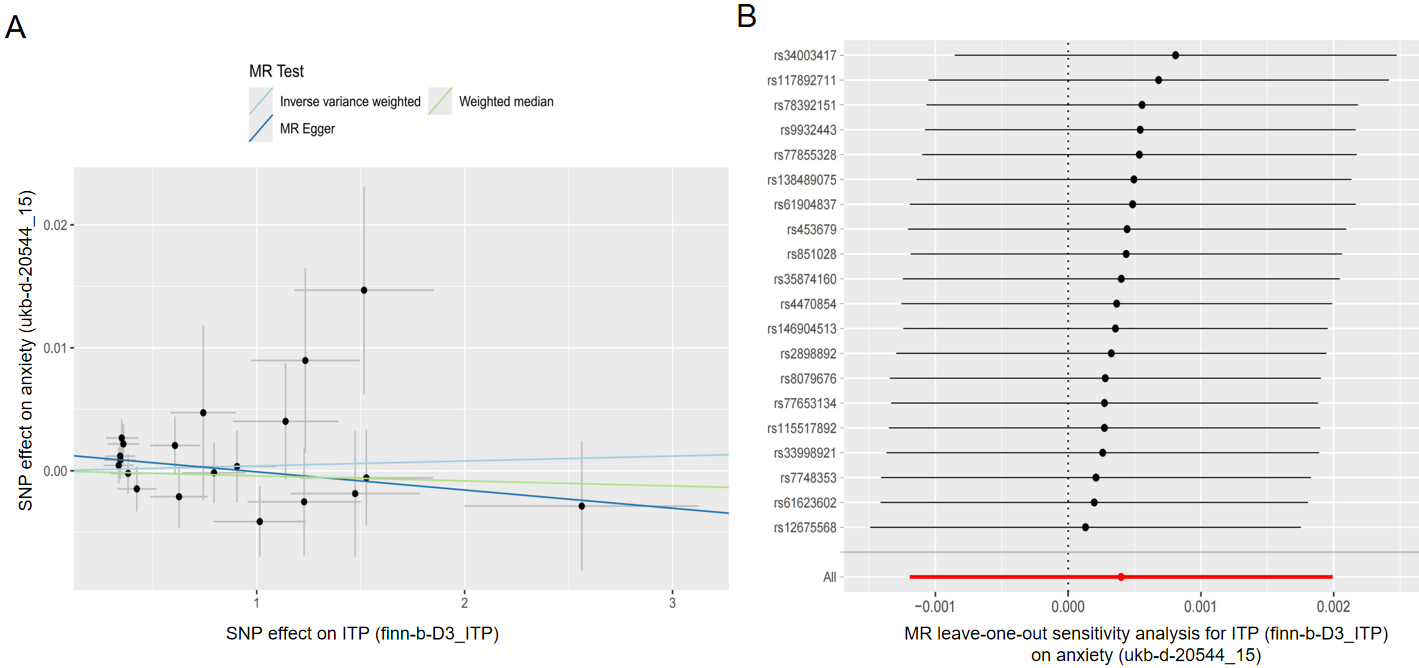
**

**Figure S1. MR analysis of ITP on anxiety**

(A) The scatter plot of primary MR analysis showed the slopes of regression lines were near zero, indicating that there was no significant association between ITP and anxiety risk. (B) The leave-one-out sensitivity analysis of the effect of ITP on anxiety showed that the confidence intervals all overlapped zero, which indicated that the null association was not driven by any single influential SNP.

Abbreviations: ITP: primary immune thrombocytopenia; MR, Mendelian randomization; SNP, single-nucleotide polymorphism.


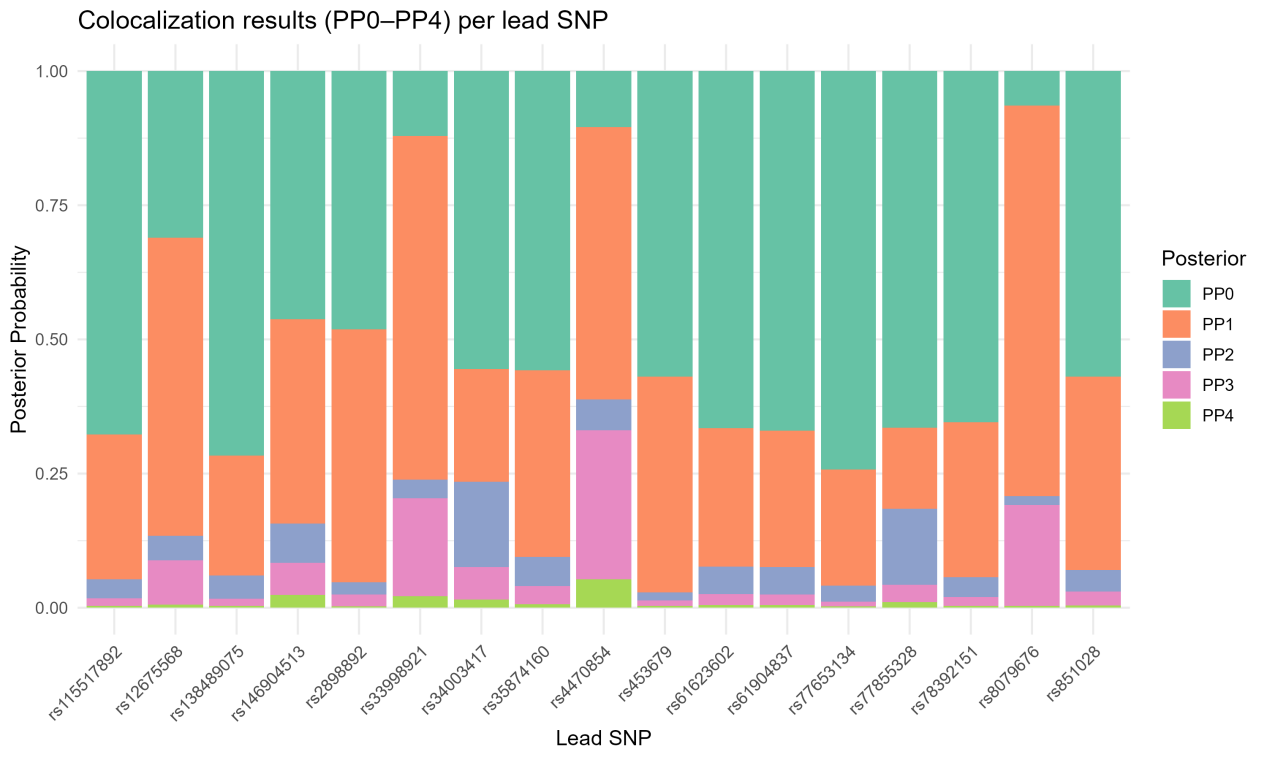


**Figure S2. Colocalization results (*PP0-PP4*) per lead SNP**

Stacked bar plot showing the posterior probabilities (*PP0-PP4*) for each of the 17 loci. PP0 indicates no association in the region; *PP1*, association with ITP only; *PP2,* association with MDD only; *PP3,* independent signals in both traits; *PP4*, shared causal variant. Across all loci, PP4 values remained very low.

**Supplementary tables**

**Table S1. Characteristics and strength metrics of genetic instruments for ITP**

| SNP | *β* exposure | *SE* exposure | *Eaf*.exposure | *R^2^* | *F* |
| --- | --- | --- | --- | --- | --- |
| rs453679 | 0.7946 | 0.1501 | 0.0718 | 0.013% | 28.024 |
| rs33998921 | -0.6068 | 0.1188 | 0.8876 | 0.012% | 26.089 |
| rs35874160 | 0.9053 | 0.1855 | 0.0462 | 0.011% | 23.818 |
| rs61904837 | 1.5274 | 0.3198 | 0.0163 | 0.011% | 22.811 |
| rs138489075 | 1.4734 | 0.3096 | 0.0174 | 0.010% | 22.648 |
| rs77653134 | 1.2336 | 0.2599 | 0.0254 | 0.010% | 22.529 |
| rs8079676 | -0.3433 | 0.0725 | 0.5688 | 0.010% | 22.422 |
| rs7748353 | 0.3588 | 0.0758 | 0.6149 | 0.010% | 22.406 |
| rs146904513 | 0.7429 | 0.1574 | 0.0644 | 0.010% | 22.277 |
| rs4470854 | 0.3363 | 0.0719 | 0.5054 | 0.010% | 21.877 |
| rs34003417 | 1.0147 | 0.2220 | 0.0324 | 0.010% | 20.891 |
| rs78392151 | 0.6264 | 0.1373 | 0.0787 | 0.010% | 20.814 |
| rs851028 | 0.3814 | 0.0837 | 0.2532 | 0.010% | 20.764 |
| rs117924460 | 1.0517 | 0.2309 | 0.0314 | 0.010% | 20.746 |
| rs12675568 | 0.3510 | 0.0771 | 0.5937 | 0.010% | 20.726 |
| rs117283193 | 1.5898 | 0.3492 | 0.0149 | 0.010% | 20.727 |
| rs61623602 | 1.5164 | 0.3333 | 0.0154 | 0.010% | 20.699 |
| rs117892711 | 2.5634 | 0.5638 | 0.0061 | 0.010% | 20.672 |
| rs77855328 | 1.2281 | 0.2714 | 0.0219 | 0.009% | 20.476 |
| rs2898892 | -0.3440 | 0.0762 | 0.3370 | 0.009% | 20.380 |
| rs9932443 | -0.4234 | 0.0939 | 0.1940 | 0.009% | 20.332 |
| rs115517892 | 1.1388 | 0.2530 | 0.0254 | 0.009% | 20.261 |
| rs6773857 | 0.3766 | 0.0852 | 0.7412 | 0.009% | 19.538 |
| Summary  (N = 23) |  |  |  | Total R² =  0.0023 | Mean F = 21.82  (19.54–28.02) |

**Table S2. Detailed SNP information of ITP on depression**

| SNP | Effect allele | Other allele | *β* | *SE* | *F* | *P* | Palindromic | MR_keep |
| --- | --- | --- | --- | --- | --- | --- | --- | --- |
| rs115517892 | A | G | 1.139 | 0.253 | 20.261 | 0.728 | FALSE | TRUE |
| rs117283193 | C | G | 1.590 | 0.349 | 20.727 | 0.863 | TRUE | FALSE |
| rs117924460 | G | C | 1.052 | 0.231 | 20.746 | 0.999 | TRUE | FALSE |
| rs12675568 | A | G | 0.351 | 0.077 | 20.726 | 0.311 | FALSE | TRUE |
| rs138489075 | C | T | 1.473 | 0.310 | 22.648 | 0.506 | FALSE | TRUE |
| rs146904513 | A | C | 0.743 | 0.157 | 22.277 | 0.117 | FALSE | TRUE |
| rs2898892 | C | T | -0.344 | 0.076 | 20.380 | 0.792 | FALSE | TRUE |
| rs33998921 | T | C | -0.607 | 0.119 | 26.089 | 0.020 | FALSE | TRUE |
| rs34003417 | A | G | 1.015 | 0.222 | 20.891 | 0.024 | FALSE | TRUE |
| rs35874160 | T | C | 0.905 | 0.186 | 23.818 | 0.880 | FALSE | TRUE |
| rs4470854 | G | T | 0.336 | 0.072 | 21.877 | 0.008 | FALSE | TRUE |
| rs453679 | C | T | 0.795 | 0.150 | 28.024 | 0.435 | FALSE | TRUE |
| rs61623602 | A | G | 1.516 | 0.333 | 20.699 | 0.926 | FALSE | TRUE |
| rs61904837 | T | G | 1.527 | 0.320 | 22.811 | 0.389 | FALSE | TRUE |
| rs77653134 | C | T | 1.234 | 0.260 | 22.529 | 0.447 | FALSE | TRUE |
| rs77855328 | T | C | 1.228 | 0.271 | 20.476 | 0.497 | FALSE | TRUE |
| rs78392151 | T | C | 0.626 | 0.137 | 20.814 | 0.879 | FALSE | TRUE |
| rs8079676 | G | A | -0.343 | 0.073 | 22.422 | 0.915 | FALSE | TRUE |
| rs851028 | G | T | 0.381 | 0.084 | 20.764 | 0.977 | FALSE | TRUE |

**Table S3. Detailed SNP information of ITP on anxiety**

| SNP | Effect allele | Other allele | *β* | *SE* | *F* | *P* | Palindromic | MR_keep |
| --- | --- | --- | --- | --- | --- | --- | --- | --- |
| rs115517892 | A | G | 1.139 | 0.253 | 20.261 | 0.393 | FALSE | TRUE |
| rs117283193 | C | G | 1.590 | 0.349 | 20.727 | 0.408 | TRUE | FALSE |
| rs117892711 | T | C | 2.563 | 0.564 | 20.672 | 0.585 | FALSE | TRUE |
| rs117924460 | G | C | 1.052 | 0.231 | 20.746 | 0.972 | TRUE | FALSE |
| rs12675568 | A | G | 0.351 | 0.077 | 20.726 | 0.076 | FALSE | TRUE |
| rs138489075 | C | T | 1.473 | 0.310 | 22.648 | 0.717 | FALSE | TRUE |
| rs146904513 | A | C | 0.743 | 0.157 | 22.277 | 0.507 | FALSE | TRUE |
| rs2898892 | C | T | -0.344 | 0.076 | 20.380 | 0.567 | FALSE | TRUE |
| rs33998921 | T | C | -0.607 | 0.119 | 26.089 | 0.379 | FALSE | TRUE |
| rs34003417 | A | G | 1.015 | 0.222 | 20.891 | 0.146 | FALSE | TRUE |
| rs35874160 | T | C | 0.905 | 0.186 | 23.818 | 0.904 | FALSE | TRUE |
| rs4470854 | G | T | 0.336 | 0.072 | 21.877 | 0.761 | FALSE | TRUE |
| rs453679 | C | T | 0.795 | 0.150 | 28.024 | 0.947 | FALSE | TRUE |
| rs61623602 | A | G | 1.516 | 0.333 | 20.699 | 0.081 | FALSE | TRUE |
| rs61904837 | T | G | 1.527 | 0.320 | 22.811 | 0.884 | FALSE | TRUE |
| rs6773857 | A | T | 0.377 | 0.085 | 19.538 | 0.561 | TRUE | FALSE |
| rs7748353 | T | G | 0.359 | 0.076 | 22.406 | 0.178 | FALSE | TRUE |
| rs77653134 | C | T | 1.234 | 0.260 | 22.529 | 0.231 | FALSE | TRUE |
| rs77855328 | T | C | 1.228 | 0.271 | 20.476 | 0.560 | FALSE | TRUE |
| rs78392151 | T | C | 0.626 | 0.137 | 20.814 | 0.406 | FALSE | TRUE |
| rs8079676 | G | A | -0.343 | 0.073 | 22.422 | 0.407 | FALSE | TRUE |
| rs851028 | G | T | 0.381 | 0.084 | 20.764 | 0.893 | FALSE | TRUE |
| rs9932443 | A | G | -0.423 | 0.094 | 20.332 | 0.418 | FALSE | TRUE |

**Table S4. Sensitivity analyses using pleiotropy-robust Mendelian randomization models**

| Method | Model Feature | *β* (*theta*) | *SE* | OR (95% CI) | *P* | *π₀* (valid IV proportion) | *σ²* (pleiotropy variance) | Interpretation |
| --- | --- | --- | --- | --- | --- | --- | --- | --- |
| MR-Corr | Accounts for correlated pleiotropy | −4.0×10⁻⁴ | 3.61×10⁻⁴ | 1.000 (0.999–1.001) | 0.264 | — | — | No significant causal effect; direction consistent with IVW |
| MRMix | Mixture model for invalid IVs | 0 | 0.00055 | 1.000 (0.999–1.001) | 1 | 0.972 | 1.24×10⁻⁵ | No significant effect; 97.2% IVs valid; minimal pleiotropy |

### *π₀*, estimated proportion of valid instruments; *σ²*, estimated residual heterogeneity variance.

**Table S5. Detailed SNP information of MDD on ITP**

| SNP | Effect allele | Other allele | *β* | *SE* | *F* | *P* | Palindromic | MR_keep |
| --- | --- | --- | --- | --- | --- | --- | --- | --- |
| rs9625613 | A | G | 0.051 | 0.012 | 19.482 | 0.570 | FALSE | TRUE |
| rs204054 | C | T | 0.027 | 0.006 | 19.531 | 0.876 | FALSE | TRUE |
| rs59746564 | C | T | -0.028 | 0.006 | 19.674 | 0.864 | FALSE | TRUE |
| rs6429297 | G | A | 0.019 | 0.004 | 19.730 | 0.780 | FALSE | TRUE |
| rs4719771 | C | T | 0.030 | 0.007 | 19.783 | 0.277 | FALSE | TRUE |
| rs1508526 | G | A | -0.021 | 0.005 | 19.877 | 0.036 | FALSE | TRUE |
| rs537362 | C | G | -0.033 | 0.007 | 19.887 | 0.136 | TRUE | FALSE |
| rs13409834 | G | A | -0.019 | 0.004 | 19.937 | 0.375 | FALSE | TRUE |
| rs28414765 | T | G | 0.019 | 0.004 | 19.937 | 0.955 | FALSE | TRUE |
| rs9564291 | G | A | 0.034 | 0.008 | 20.014 | 0.245 | FALSE | TRUE |
| rs2972166 | A | G | -0.022 | 0.005 | 20.063 | 0.389 | FALSE | TRUE |
| rs908639 | C | T | 0.024 | 0.005 | 20.084 | 0.741 | FALSE | TRUE |
| rs73334050 | G | C | -0.038 | 0.008 | 20.143 | 0.731 | TRUE | FALSE |
| rs73350816 | A | G | -0.027 | 0.006 | 20.174 | 0.934 | FALSE | TRUE |
| rs2981329 | T | C | -0.023 | 0.005 | 20.250 | 0.835 | FALSE | TRUE |
| rs115284696 | T | C | -0.050 | 0.011 | 20.291 | 0.917 | FALSE | TRUE |
| rs12129780 | T | C | 0.033 | 0.007 | 20.312 | 0.897 | FALSE | TRUE |
| rs62419352 | G | A | 0.063 | 0.014 | 20.314 | 0.852 | FALSE | TRUE |
| rs61876142 | A | T | 0.028 | 0.006 | 20.324 | 0.073 | TRUE | FALSE |
| rs11826373 | C | T | 0.025 | 0.006 | 20.332 | 0.368 | FALSE | TRUE |
| rs7926849 | T | C | 0.019 | 0.004 | 20.355 | 0.956 | FALSE | TRUE |
| rs74927570 | G | T | 0.025 | 0.006 | 20.411 | 0.066 | FALSE | TRUE |
| rs12513440 | A | G | 0.023 | 0.005 | 20.430 | 0.222 | FALSE | TRUE |
| rs58250641 | C | T | -0.024 | 0.005 | 20.506 | 0.083 | FALSE | TRUE |
| rs12506416 | T | C | 0.031 | 0.007 | 20.516 | 0.318 | FALSE | TRUE |
| rs1442129 | G | A | 0.020 | 0.004 | 20.565 | 0.001 | FALSE | TRUE |
| rs56115484 | T | C | -0.025 | 0.005 | 20.585 | 0.978 | FALSE | TRUE |
| rs10931670 | G | T | 0.022 | 0.005 | 20.627 | 0.328 | FALSE | TRUE |
| rs12420321 | C | G | 0.024 | 0.005 | 20.677 | 0.572 | TRUE | FALSE |
| rs75889584 | G | A | 0.092 | 0.020 | 20.698 | 0.741 | FALSE | TRUE |
| rs737022 | A | G | -0.020 | 0.004 | 20.777 | 0.216 | FALSE | TRUE |
| rs12188637 | G | A | -0.022 | 0.005 | 20.816 | 0.072 | FALSE | TRUE |
| rs905111 | G | A | -0.022 | 0.005 | 20.816 | 0.324 | FALSE | TRUE |
| rs4735240 | T | C | 0.020 | 0.004 | 20.868 | 0.318 | FALSE | TRUE |
| rs76923051 | G | A | -0.060 | 0.013 | 20.868 | 0.070 | FALSE | TRUE |
| rs779 | A | G | -0.031 | 0.007 | 20.917 | 0.368 | FALSE | TRUE |
| rs66651392 | T | C | 0.023 | 0.005 | 20.976 | 0.908 | FALSE | TRUE |
| rs56211578 | C | A | 0.028 | 0.006 | 21.007 | 0.399 | FALSE | TRUE |
| rs138039555 | T | C | -0.039 | 0.008 | 21.007 | 0.567 | FALSE | TRUE |
| rs7631372 | T | C | -0.026 | 0.006 | 21.062 | 0.294 | FALSE | TRUE |
| rs111750830 | A | G | 0.036 | 0.008 | 21.066 | 0.881 | FALSE | TRUE |
| rs5771864 | C | T | -0.020 | 0.004 | 21.076 | 0.911 | FALSE | TRUE |
| rs7499405 | A | G | -0.020 | 0.004 | 21.076 | 0.424 | FALSE | TRUE |
| rs4707630 | T | C | 0.023 | 0.005 | 21.085 | 0.014 | FALSE | TRUE |
| rs67767662 | G | T | 0.025 | 0.005 | 21.092 | 0.029 | FALSE | TRUE |
| rs7098208 | T | C | 0.024 | 0.005 | 21.125 | 0.000 | FALSE | FALSE |
| rs1466386 | G | A | -0.026 | 0.006 | 21.128 | 0.045 | FALSE | TRUE |
| rs12125521 | C | T | -0.022 | 0.005 | 21.198 | 0.194 | FALSE | TRUE |
| rs1359815 | T | C | 0.022 | 0.005 | 21.198 | 0.239 | FALSE | TRUE |
| rs11755188 | T | C | 0.043 | 0.009 | 21.279 | 0.251 | FALSE | TRUE |
| rs1477272 | C | T | -0.020 | 0.004 | 21.286 | 0.510 | FALSE | TRUE |
| rs34751496 | A | G | -0.020 | 0.004 | 21.286 | 0.547 | FALSE | TRUE |
| rs52849 | G | A | -0.051 | 0.011 | 21.328 | 0.495 | FALSE | TRUE |
| rs10415547 | C | T | 0.023 | 0.005 | 21.344 | 0.724 | FALSE | TRUE |
| rs11662323 | T | C | -0.025 | 0.005 | 21.369 | 0.249 | FALSE | TRUE |
| rs75520450 | C | T | -0.053 | 0.012 | 21.401 | 0.003 | FALSE | FALSE |
| rs6602459 | C | G | -0.024 | 0.005 | 21.413 | 0.717 | TRUE | FALSE |
| rs112610187 | T | G | -0.048 | 0.010 | 21.447 | 0.374 | FALSE | TRUE |
| rs36168783 | T | C | -0.020 | 0.004 | 21.496 | 0.580 | FALSE | TRUE |
| rs4404022 | T | A | 0.020 | 0.004 | 21.496 | 0.040 | TRUE | FALSE |
| rs751996 | C | G | -0.020 | 0.004 | 21.496 | 0.931 | TRUE | FALSE |
| rs111605708 | T | A | -0.052 | 0.011 | 21.503 | 0.816 | TRUE | FALSE |
| rs12615271 | A | G | -0.022 | 0.005 | 21.584 | 0.373 | FALSE | TRUE |
| rs6832890 | C | G | -0.025 | 0.005 | 21.605 | 0.478 | TRUE | FALSE |
| rs34947393 | C | G | -0.061 | 0.013 | 21.658 | 0.683 | TRUE | FALSE |
| rs6449561 | C | G | 0.024 | 0.005 | 21.658 | 0.019 | TRUE | FALSE |
| rs79780963 | T | C | -0.037 | 0.008 | 21.699 | 0.945 | FALSE | TRUE |
| rs12979771 | T | C | 0.022 | 0.005 | 21.712 | 0.167 | FALSE | TRUE |
| rs80243771 | G | A | 0.037 | 0.008 | 21.739 | 0.482 | FALSE | TRUE |
| rs71524105 | A | G | 0.033 | 0.007 | 21.866 | 0.954 | FALSE | TRUE |
| rs2237738 | G | A | -0.021 | 0.004 | 21.919 | 0.104 | FALSE | TRUE |
| rs187843156 | T | C | 0.030 | 0.006 | 21.973 | 0.417 | FALSE | TRUE |
| rs11078936 | C | T | -0.021 | 0.005 | 21.986 | 0.172 | FALSE | TRUE |
| rs7584419 | A | C | 0.021 | 0.005 | 21.986 | 0.225 | FALSE | TRUE |
| rs12627769 | A | G | -0.026 | 0.006 | 22.005 | 0.105 | FALSE | TRUE |
| rs12487069 | A | G | -0.020 | 0.004 | 22.068 | 0.160 | FALSE | TRUE |
| rs547488 | C | G | 0.020 | 0.004 | 22.068 | 0.562 | TRUE | FALSE |
| rs9920233 | A | C | -0.020 | 0.004 | 22.068 | 0.400 | FALSE | TRUE |
| rs1530310 | G | A | -0.022 | 0.005 | 22.110 | 0.684 | FALSE | TRUE |
| rs55874815 | C | A | -0.048 | 0.010 | 22.118 | 0.631 | FALSE | TRUE |
| rs10747480 | A | G | 0.021 | 0.004 | 22.133 | 0.174 | FALSE | TRUE |
| rs12542837 | C | T | 0.021 | 0.004 | 22.133 | 0.534 | FALSE | TRUE |
| rs10838721 | G | A | 0.065 | 0.014 | 22.137 | 0.412 | FALSE | TRUE |
| rs34341246 | C | T | 0.020 | 0.004 | 22.287 | 0.194 | FALSE | TRUE |
| rs72830861 | T | C | 0.057 | 0.012 | 22.404 | 0.755 | FALSE | TRUE |
| rs1955596 | C | T | 0.040 | 0.008 | 22.450 | 0.882 | FALSE | TRUE |
| rs2292996 | C | T | 0.020 | 0.004 | 22.507 | 0.442 | FALSE | TRUE |
| rs9825208 | T | G | -0.020 | 0.004 | 22.507 | 0.811 | FALSE | TRUE |
| rs6846784 | C | T | 0.021 | 0.004 | 22.563 | 0.424 | FALSE | TRUE |
| rs2888566 | C | T | 0.029 | 0.006 | 22.563 | 0.174 | FALSE | TRUE |
| rs353308 | A | T | 0.038 | 0.008 | 22.681 | 0.709 | TRUE | FALSE |
| rs185214 | A | G | -0.024 | 0.005 | 22.702 | 0.722 | FALSE | TRUE |
| rs12335840 | T | C | 0.027 | 0.006 | 22.732 | 0.595 | FALSE | TRUE |
| rs7046881 | G | T | -0.021 | 0.004 | 22.779 | 0.744 | FALSE | TRUE |
| rs56080343 | C | T | 0.027 | 0.006 | 22.903 | 0.029 | FALSE | TRUE |
| rs150586937 | A | C | -0.107 | 0.022 | 22.989 | 0.664 | FALSE | TRUE |
| rs55993110 | C | G | -0.028 | 0.006 | 23.007 | 0.412 | TRUE | FALSE |
| rs6553965 | G | A | 0.022 | 0.005 | 23.291 | 0.548 | FALSE | TRUE |
| rs11220798 | T | C | 0.040 | 0.008 | 23.342 | 0.373 | FALSE | TRUE |
| rs3783007 | G | T | 0.021 | 0.004 | 23.434 | 0.946 | FALSE | TRUE |
| rs4350019 | G | A | 0.029 | 0.006 | 23.498 | 0.018 | FALSE | TRUE |
| rs4971040 | C | A | 0.029 | 0.006 | 23.498 | 0.391 | FALSE | TRUE |
| rs76134318 | T | G | 0.048 | 0.010 | 23.592 | 0.202 | FALSE | TRUE |
| rs6746122 | A | G | 0.023 | 0.005 | 24.138 | 0.820 | FALSE | TRUE |
| rs1512547 | A | G | -0.023 | 0.005 | 24.156 | 0.673 | FALSE | TRUE |
| rs9642819 | A | G | 0.024 | 0.005 | 24.190 | 0.643 | FALSE | TRUE |
| rs7097344 | T | C | 0.073 | 0.015 | 24.267 | 0.698 | FALSE | TRUE |
| rs261909 | C | G | 0.021 | 0.004 | 24.307 | 0.293 | TRUE | FALSE |
| rs8050761 | G | C | -0.022 | 0.005 | 24.338 | 0.031 | TRUE | FALSE |
| rs2784738 | A | G | 0.025 | 0.005 | 24.404 | 0.618 | FALSE | TRUE |
| rs55908506 | C | A | -0.040 | 0.008 | 24.626 | 0.417 | FALSE | TRUE |
| rs1862743 | A | C | -0.021 | 0.004 | 24.768 | 0.071 | FALSE | TRUE |
| rs4818766 | G | A | 0.022 | 0.005 | 24.778 | 0.567 | FALSE | TRUE |
| rs4895718 | T | C | -0.022 | 0.005 | 24.778 | 0.370 | FALSE | TRUE |
| rs12661596 | G | A | -0.025 | 0.005 | 24.804 | 0.226 | FALSE | TRUE |
| rs76227152 | C | A | -0.052 | 0.010 | 24.808 | 0.168 | FALSE | TRUE |
| rs10821866 | A | C | -0.022 | 0.004 | 25.000 | 0.485 | FALSE | TRUE |
| rs10961649 | T | C | 0.023 | 0.005 | 25.000 | 0.117 | FALSE | TRUE |
| rs60157091 | T | C | 0.022 | 0.004 | 25.000 | 0.341 | FALSE | TRUE |
| rs9538160 | A | G | -0.023 | 0.005 | 25.000 | 0.443 | FALSE | TRUE |
| rs1116690 | G | A | 0.025 | 0.005 | 25.000 | 0.172 | FALSE | TRUE |
| rs6479487 | G | T | 0.030 | 0.006 | 25.170 | 0.853 | FALSE | TRUE |
| rs77579223 | T | C | -0.025 | 0.005 | 25.200 | 0.145 | FALSE | TRUE |
| rs1016165 | C | T | -0.022 | 0.004 | 25.228 | 0.120 | FALSE | TRUE |
| rs1908953 | G | T | 0.022 | 0.004 | 25.457 | 0.243 | FALSE | TRUE |
| rs2582954 | T | C | -0.022 | 0.004 | 25.457 | 0.213 | FALSE | TRUE |
| rs34416809 | C | T | -0.044 | 0.009 | 25.457 | 0.022 | FALSE | TRUE |
| rs28649197 | G | T | -0.074 | 0.015 | 25.482 | 0.521 | FALSE | TRUE |
| rs56101042 | G | A | 0.028 | 0.006 | 25.548 | 0.710 | FALSE | TRUE |
| rs578174 | G | A | -0.036 | 0.007 | 25.559 | 0.491 | FALSE | TRUE |
| rs12512642 | T | C | 0.026 | 0.005 | 25.580 | 0.470 | FALSE | TRUE |
| rs6589675 | G | C | -0.035 | 0.007 | 25.583 | 0.972 | TRUE | FALSE |
| rs2132342 | A | G | -0.022 | 0.004 | 25.686 | 0.973 | FALSE | TRUE |
| rs112844822 | T | C | 0.065 | 0.013 | 25.867 | 0.341 | FALSE | TRUE |
| rs2246221 | A | G | 0.022 | 0.004 | 25.939 | 0.102 | FALSE | TRUE |
| rs10896012 | C | T | 0.027 | 0.005 | 25.952 | 0.721 | FALSE | TRUE |
| rs34488670 | C | T | 0.027 | 0.005 | 25.952 | 0.919 | FALSE | TRUE |
| rs1414622 | C | T | -0.041 | 0.008 | 26.010 | 0.131 | FALSE | TRUE |
| rs6765468 | A | G | 0.024 | 0.005 | 26.099 | 0.517 | FALSE | TRUE |
| rs7842361 | T | C | -0.033 | 0.006 | 26.612 | 0.490 | FALSE | TRUE |
| rs4746612 | C | T | -0.023 | 0.004 | 26.616 | 0.010 | FALSE | TRUE |
| rs12160976 | A | G | -0.024 | 0.005 | 26.731 | 0.403 | FALSE | TRUE |
| rs2509805 | C | T | -0.024 | 0.005 | 26.995 | 0.423 | FALSE | TRUE |
| rs780025 | T | G | 0.024 | 0.005 | 26.995 | 0.580 | FALSE | TRUE |
| rs884285 | T | C | 0.024 | 0.005 | 26.995 | 0.755 | FALSE | TRUE |
| rs7758630 | A | T | -0.023 | 0.004 | 27.087 | 0.013 | TRUE | FALSE |
| rs12526217 | C | T | -0.034 | 0.007 | 27.166 | 0.518 | FALSE | TRUE |
| rs6606679 | C | T | 0.024 | 0.005 | 27.221 | 0.422 | FALSE | TRUE |
| rs2861885 | A | C | 0.035 | 0.007 | 27.289 | 0.962 | FALSE | TRUE |
| rs4653218 | C | T | -0.023 | 0.004 | 27.324 | 0.063 | FALSE | TRUE |
| rs10755215 | T | C | 0.023 | 0.004 | 27.380 | 0.570 | FALSE | TRUE |
| rs3905238 | G | A | -0.024 | 0.005 | 27.504 | 0.161 | FALSE | TRUE |
| rs7515828 | C | T | -0.023 | 0.004 | 27.563 | 0.133 | FALSE | TRUE |
| rs4776768 | C | T | 0.025 | 0.005 | 27.563 | 0.551 | FALSE | TRUE |
| rs11250015 | G | A | -0.029 | 0.006 | 27.994 | 0.829 | FALSE | TRUE |
| rs12589834 | G | A | -0.027 | 0.005 | 28.028 | 0.622 | FALSE | TRUE |
| rs10835380 | G | A | -0.024 | 0.005 | 28.136 | 0.635 | FALSE | TRUE |
| rs7595982 | G | T | 0.025 | 0.005 | 28.367 | 0.055 | FALSE | TRUE |
| rs10061069 | C | G | -0.028 | 0.005 | 28.994 | 0.238 | TRUE | FALSE |
| rs72737052 | G | A | 0.047 | 0.009 | 29.185 | 0.517 | FALSE | TRUE |
| rs12619197 | A | G | 0.023 | 0.004 | 29.361 | 0.794 | FALSE | TRUE |
| rs3793577 | G | A | 0.023 | 0.004 | 29.614 | 0.077 | FALSE | TRUE |
| rs699927 | G | T | 0.024 | 0.004 | 29.752 | 0.793 | FALSE | TRUE |
| rs7200826 | T | C | 0.027 | 0.005 | 29.914 | 0.364 | FALSE | TRUE |
| rs59082935 | T | C | 0.036 | 0.007 | 30.250 | 0.940 | FALSE | TRUE |
| rs508502 | T | C | -0.026 | 0.005 | 30.250 | 0.835 | FALSE | TRUE |
| rs10235664 | C | T | -0.027 | 0.005 | 30.362 | 0.334 | FALSE | TRUE |
| rs247910 | G | A | 0.024 | 0.004 | 30.378 | 0.358 | FALSE | TRUE |
| rs4730387 | A | T | 0.024 | 0.004 | 30.635 | 0.330 | TRUE | FALSE |
| rs9536381 | T | C | 0.026 | 0.005 | 30.730 | 0.913 | FALSE | TRUE |
| rs9364755 | G | A | 0.028 | 0.005 | 30.792 | 0.031 | FALSE | TRUE |
| rs76954012 | A | T | 0.041 | 0.007 | 30.998 | 0.140 | TRUE | FALSE |
| rs59283172 | A | G | -0.039 | 0.007 | 31.041 | 0.799 | FALSE | TRUE |
| rs2522831 | C | T | 0.024 | 0.004 | 31.152 | 0.344 | FALSE | TRUE |
| rs28541419 | G | C | -0.029 | 0.005 | 31.533 | 0.131 | TRUE | FALSE |
| rs9831648 | T | G | -0.029 | 0.005 | 31.533 | 0.067 | FALSE | TRUE |
| rs198457 | T | C | -0.032 | 0.006 | 31.641 | 0.397 | FALSE | TRUE |
| rs843812 | A | G | 0.025 | 0.004 | 31.769 | 0.095 | FALSE | TRUE |
| rs72948506 | A | G | 0.027 | 0.005 | 31.790 | 0.463 | FALSE | TRUE |
| rs17641524 | T | C | -0.030 | 0.005 | 32.040 | 0.052 | FALSE | TRUE |
| rs2111592 | A | G | 0.026 | 0.005 | 32.689 | 0.838 | FALSE | TRUE |
| rs61914045 | A | G | 0.031 | 0.005 | 32.744 | 0.797 | FALSE | TRUE |
| rs4141983 | C | T | -0.026 | 0.005 | 32.938 | 0.911 | FALSE | TRUE |
| rs2214123 | G | A | -0.026 | 0.005 | 33.640 | 0.872 | FALSE | TRUE |
| rs10913112 | T | C | -0.026 | 0.005 | 33.898 | 0.749 | FALSE | TRUE |
| rs7538938 | C | T | 0.025 | 0.004 | 34.073 | 0.165 | FALSE | TRUE |
| rs62535714 | A | G | 0.034 | 0.006 | 34.162 | 0.197 | FALSE | TRUE |
| rs2418449 | C | T | -0.028 | 0.005 | 34.271 | 0.667 | FALSE | TRUE |
| rs150186873 | C | A | 0.070 | 0.012 | 34.418 | 0.397 | FALSE | TRUE |
| rs1367635 | C | T | 0.025 | 0.004 | 34.618 | 0.367 | FALSE | TRUE |
| rs12919291 | C | G | 0.033 | 0.006 | 35.348 | 0.292 | TRUE | FALSE |
| rs7241572 | A | G | 0.032 | 0.005 | 35.778 | 0.642 | FALSE | TRUE |
| rs354155 | C | G | -0.045 | 0.008 | 35.840 | 0.019 | TRUE | FALSE |
| rs7152906 | C | T | 0.026 | 0.004 | 36.000 | 0.212 | FALSE | TRUE |
| rs2876520 | G | C | 0.026 | 0.004 | 36.560 | 0.666 | TRUE | FALSE |
| rs66511648 | C | T | 0.030 | 0.005 | 38.285 | 0.821 | FALSE | TRUE |
| rs9529218 | T | C | -0.034 | 0.005 | 39.643 | 0.986 | FALSE | TRUE |
| rs4936276 | C | G | 0.028 | 0.004 | 39.919 | 0.861 | TRUE | FALSE |
| rs13037326 | T | C | 0.031 | 0.005 | 40.025 | 0.425 | FALSE | TRUE |
| rs4799949 | T | C | -0.029 | 0.005 | 40.295 | 0.946 | FALSE | TRUE |
| rs754287 | A | T | -0.029 | 0.005 | 41.245 | 0.339 | TRUE | FALSE |
| rs150346963 | T | C | 0.028 | 0.004 | 41.368 | 0.043 | FALSE | TRUE |
| rs7551758 | G | T | 0.028 | 0.004 | 43.315 | 0.209 | FALSE | TRUE |
| rs4497414 | C | T | 0.029 | 0.004 | 43.740 | 0.184 | FALSE | TRUE |
| rs1021363 | G | A | -0.030 | 0.005 | 44.444 | 0.278 | FALSE | TRUE |
| rs1931388 | G | A | -0.030 | 0.004 | 44.951 | 0.247 | FALSE | TRUE |
| rs7725715 | A | G | 0.029 | 0.004 | 45.484 | 0.506 | FALSE | TRUE |
| rs1950829 | G | A | -0.030 | 0.004 | 47.706 | 0.858 | FALSE | TRUE |
| rs3807865 | A | G | 0.031 | 0.004 | 49.638 | 0.708 | FALSE | TRUE |
| rs12967143 | C | G | -0.035 | 0.005 | 53.882 | 0.591 | TRUE | FALSE |
| rs30266 | A | G | 0.037 | 0.005 | 63.306 | 0.425 | FALSE | TRUE |
| rs2568958 | A | G | 0.038 | 0.004 | 75.374 | 0.356 | FALSE | TRUE |
| rs2232423 | G | A | -0.062 | 0.007 | 78.449 | 0.682 | FALSE | TRUE |
| rs7595982 | G | T | 0.025 | 0.005 | 28.367 | 0.055 | FALSE | TRUE |
| rs10061069 | C | G | -0.028 | 0.005 | 28.994 | 0.238 | TRUE | FALSE |
| rs72737052 | G | A | 0.047 | 0.009 | 29.185 | 0.517 | FALSE | TRUE |
| rs12619197 | A | G | 0.023 | 0.004 | 29.361 | 0.794 | FALSE | TRUE |
| rs3793577 | G | A | 0.023 | 0.004 | 29.614 | 0.077 | FALSE | TRUE |
| rs699927 | G | T | 0.024 | 0.004 | 29.752 | 0.793 | FALSE | TRUE |
| rs7200826 | T | C | 0.027 | 0.005 | 29.914 | 0.364 | FALSE | TRUE |
| rs59082935 | T | C | 0.036 | 0.007 | 30.250 | 0.940 | FALSE | TRUE |
| rs508502 | T | C | -0.026 | 0.005 | 30.250 | 0.835 | FALSE | TRUE |
| rs10235664 | C | T | -0.027 | 0.005 | 30.362 | 0.334 | FALSE | TRUE |
| rs247910 | G | A | 0.024 | 0.004 | 30.378 | 0.358 | FALSE | TRUE |
| rs4730387 | A | T | 0.024 | 0.004 | 30.635 | 0.330 | TRUE | FALSE |
| rs9536381 | T | C | 0.026 | 0.005 | 30.730 | 0.913 | FALSE | TRUE |
| rs9364755 | G | A | 0.028 | 0.005 | 30.792 | 0.031 | FALSE | TRUE |
| rs76954012 | A | T | 0.041 | 0.007 | 30.998 | 0.140 | TRUE | FALSE |
| rs59283172 | A | G | -0.039 | 0.007 | 31.041 | 0.799 | FALSE | TRUE |
| rs2522831 | C | T | 0.024 | 0.004 | 31.152 | 0.344 | FALSE | TRUE |
| rs28541419 | G | C | -0.029 | 0.005 | 31.533 | 0.131 | TRUE | FALSE |
| rs9831648 | T | G | -0.029 | 0.005 | 31.533 | 0.067 | FALSE | TRUE |
| rs198457 | T | C | -0.032 | 0.006 | 31.641 | 0.397 | FALSE | TRUE |
| rs843812 | A | G | 0.025 | 0.004 | 31.769 | 0.095 | FALSE | TRUE |
| rs72948506 | A | G | 0.027 | 0.005 | 31.790 | 0.463 | FALSE | TRUE |
| rs17641524 | T | C | -0.030 | 0.005 | 32.040 | 0.052 | FALSE | TRUE |
| rs2111592 | A | G | 0.026 | 0.005 | 32.689 | 0.838 | FALSE | TRUE |
| rs61914045 | A | G | 0.031 | 0.005 | 32.744 | 0.797 | FALSE | TRUE |
| rs4141983 | C | T | -0.026 | 0.005 | 32.938 | 0.911 | FALSE | TRUE |
| rs2214123 | G | A | -0.026 | 0.005 | 33.640 | 0.872 | FALSE | TRUE |
| rs10913112 | T | C | -0.026 | 0.005 | 33.898 | 0.749 | FALSE | TRUE |
| rs7538938 | C | T | 0.025 | 0.004 | 34.073 | 0.165 | FALSE | TRUE |
| rs62535714 | A | G | 0.034 | 0.006 | 34.162 | 0.197 | FALSE | TRUE |
| rs2418449 | C | T | -0.028 | 0.005 | 34.271 | 0.667 | FALSE | TRUE |
| rs150186873 | C | A | 0.070 | 0.012 | 34.418 | 0.397 | FALSE | TRUE |
| rs1367635 | C | T | 0.025 | 0.004 | 34.618 | 0.367 | FALSE | TRUE |
| rs12919291 | C | G | 0.033 | 0.006 | 35.348 | 0.292 | TRUE | FALSE |
| rs7241572 | A | G | 0.032 | 0.005 | 35.778 | 0.642 | FALSE | TRUE |
| rs354155 | C | G | -0.045 | 0.008 | 35.840 | 0.019 | TRUE | FALSE |
| rs7152906 | C | T | 0.026 | 0.004 | 36.000 | 0.212 | FALSE | TRUE |
| rs2876520 | G | C | 0.026 | 0.004 | 36.560 | 0.666 | TRUE | FALSE |
| rs66511648 | C | T | 0.030 | 0.005 | 38.285 | 0.821 | FALSE | TRUE |
| rs9529218 | T | C | -0.034 | 0.005 | 39.643 | 0.986 | FALSE | TRUE |
| rs4936276 | C | G | 0.028 | 0.004 | 39.919 | 0.861 | TRUE | FALSE |
| rs13037326 | T | C | 0.031 | 0.005 | 40.025 | 0.425 | FALSE | TRUE |
| rs4799949 | T | C | -0.029 | 0.005 | 40.295 | 0.946 | FALSE | TRUE |
| rs754287 | A | T | -0.029 | 0.005 | 41.245 | 0.339 | TRUE | FALSE |
| rs150346963 | T | C | 0.028 | 0.004 | 41.368 | 0.043 | FALSE | TRUE |
| rs7551758 | G | T | 0.028 | 0.004 | 43.315 | 0.209 | FALSE | TRUE |
| rs4497414 | C | T | 0.029 | 0.004 | 43.740 | 0.184 | FALSE | TRUE |
| rs1021363 | G | A | -0.030 | 0.005 | 44.444 | 0.278 | FALSE | TRUE |
| rs1931388 | G | A | -0.030 | 0.004 | 44.951 | 0.247 | FALSE | TRUE |
| rs7725715 | A | G | 0.029 | 0.004 | 45.484 | 0.506 | FALSE | TRUE |
| rs1950829 | G | A | -0.030 | 0.004 | 47.706 | 0.858 | FALSE | TRUE |
| rs3807865 | A | G | 0.031 | 0.004 | 49.638 | 0.708 | FALSE | TRUE |
| rs12967143 | C | G | -0.035 | 0.005 | 53.882 | 0.591 | TRUE | FALSE |
| rs30266 | A | G | 0.037 | 0.005 | 63.306 | 0.425 | FALSE | TRUE |
| rs2568958 | A | G | 0.038 | 0.004 | 75.374 | 0.356 | FALSE | TRUE |
| rs2232423 | G | A | -0.062 | 0.007 | 78.449 | 0.682 | FALSE | TRUE |
| rs7595982 | G | T | 0.025 | 0.005 | 28.367 | 0.055 | FALSE | TRUE |
| rs10061069 | C | G | -0.028 | 0.005 | 28.994 | 0.238 | TRUE | FALSE |
| rs72737052 | G | A | 0.047 | 0.009 | 29.185 | 0.517 | FALSE | TRUE |
| rs12619197 | A | G | 0.023 | 0.004 | 29.361 | 0.794 | FALSE | TRUE |
| rs3793577 | G | A | 0.023 | 0.004 | 29.614 | 0.077 | FALSE | TRUE |
| rs699927 | G | T | 0.024 | 0.004 | 29.752 | 0.793 | FALSE | TRUE |
| rs7200826 | T | C | 0.027 | 0.005 | 29.914 | 0.364 | FALSE | TRUE |
| rs59082935 | T | C | 0.036 | 0.007 | 30.250 | 0.940 | FALSE | TRUE |
| rs508502 | T | C | -0.026 | 0.005 | 30.250 | 0.835 | FALSE | TRUE |
| rs10235664 | C | T | -0.027 | 0.005 | 30.362 | 0.334 | FALSE | TRUE |
| rs247910 | G | A | 0.024 | 0.004 | 30.378 | 0.358 | FALSE | TRUE |
| rs4730387 | A | T | 0.024 | 0.004 | 30.635 | 0.330 | TRUE | FALSE |
| rs9536381 | T | C | 0.026 | 0.005 | 30.730 | 0.913 | FALSE | TRUE |
| rs9364755 | G | A | 0.028 | 0.005 | 30.792 | 0.031 | FALSE | TRUE |
| rs76954012 | A | T | 0.041 | 0.007 | 30.998 | 0.140 | TRUE | FALSE |
| rs59283172 | A | G | -0.039 | 0.007 | 31.041 | 0.799 | FALSE | TRUE |
| rs2522831 | C | T | 0.024 | 0.004 | 31.152 | 0.344 | FALSE | TRUE |
| rs28541419 | G | C | -0.029 | 0.005 | 31.533 | 0.131 | TRUE | FALSE |
| rs9831648 | T | G | -0.029 | 0.005 | 31.533 | 0.067 | FALSE | TRUE |
| rs198457 | T | C | -0.032 | 0.006 | 31.641 | 0.397 | FALSE | TRUE |
| rs843812 | A | G | 0.025 | 0.004 | 31.769 | 0.095 | FALSE | TRUE |
| rs72948506 | A | G | 0.027 | 0.005 | 31.790 | 0.463 | FALSE | TRUE |
| rs17641524 | T | C | -0.030 | 0.005 | 32.040 | 0.052 | FALSE | TRUE |
| rs2111592 | A | G | 0.026 | 0.005 | 32.689 | 0.838 | FALSE | TRUE |
| rs61914045 | A | G | 0.031 | 0.005 | 32.744 | 0.797 | FALSE | TRUE |
| rs4141983 | C | T | -0.026 | 0.005 | 32.938 | 0.911 | FALSE | TRUE |
| rs2214123 | G | A | -0.026 | 0.005 | 33.640 | 0.872 | FALSE | TRUE |
| rs10913112 | T | C | -0.026 | 0.005 | 33.898 | 0.749 | FALSE | TRUE |
| rs7538938 | C | T | 0.025 | 0.004 | 34.073 | 0.165 | FALSE | TRUE |
| rs62535714 | A | G | 0.034 | 0.006 | 34.162 | 0.197 | FALSE | TRUE |
| rs2418449 | C | T | -0.028 | 0.005 | 34.271 | 0.667 | FALSE | TRUE |
| rs150186873 | C | A | 0.070 | 0.012 | 34.418 | 0.397 | FALSE | TRUE |
| rs1367635 | C | T | 0.025 | 0.004 | 34.618 | 0.367 | FALSE | TRUE |
| rs12919291 | C | G | 0.033 | 0.006 | 35.348 | 0.292 | TRUE | FALSE |
| rs7241572 | A | G | 0.032 | 0.005 | 35.778 | 0.642 | FALSE | TRUE |
| rs354155 | C | G | -0.045 | 0.008 | 35.840 | 0.019 | TRUE | FALSE |
| rs7152906 | C | T | 0.026 | 0.004 | 36.000 | 0.212 | FALSE | TRUE |
| rs2876520 | G | C | 0.026 | 0.004 | 36.560 | 0.666 | TRUE | FALSE |
| rs66511648 | C | T | 0.030 | 0.005 | 38.285 | 0.821 | FALSE | TRUE |
| rs9529218 | T | C | -0.034 | 0.005 | 39.643 | 0.986 | FALSE | TRUE |
| rs4936276 | C | G | 0.028 | 0.004 | 39.919 | 0.861 | TRUE | FALSE |
| rs13037326 | T | C | 0.031 | 0.005 | 40.025 | 0.425 | FALSE | TRUE |
| rs4799949 | T | C | -0.029 | 0.005 | 40.295 | 0.946 | FALSE | TRUE |
| rs754287 | A | T | -0.029 | 0.005 | 41.245 | 0.339 | TRUE | FALSE |
| rs150346963 | T | C | 0.028 | 0.004 | 41.368 | 0.043 | FALSE | TRUE |
| rs7551758 | G | T | 0.028 | 0.004 | 43.315 | 0.209 | FALSE | TRUE |
| rs4497414 | C | T | 0.029 | 0.004 | 43.740 | 0.184 | FALSE | TRUE |
| rs1021363 | G | A | -0.030 | 0.005 | 44.444 | 0.278 | FALSE | TRUE |
| rs1931388 | G | A | -0.030 | 0.004 | 44.951 | 0.247 | FALSE | TRUE |
| rs7725715 | A | G | 0.029 | 0.004 | 45.484 | 0.506 | FALSE | TRUE |
| rs1950829 | G | A | -0.030 | 0.004 | 47.706 | 0.858 | FALSE | TRUE |
| rs3807865 | A | G | 0.031 | 0.004 | 49.638 | 0.708 | FALSE | TRUE |
| rs12967143 | C | G | -0.035 | 0.005 | 53.882 | 0.591 | TRUE | FALSE |
| rs30266 | A | G | 0.037 | 0.005 | 63.306 | 0.425 | FALSE | TRUE |
| rs2568958 | A | G | 0.038 | 0.004 | 75.374 | 0.356 | FALSE | TRUE |
| rs2232423 | G | A | -0.062 | 0.007 | 78.449 | 0.682 | FALSE | TRUE |

**Table S6. The results of colocalization analysis（ITP on MDD）**

| Lead SNPs | CHR | POS | nSNPs | *PP0* | *PP1* | *PP2* | *PP3* | *PP4* |
| --- | --- | --- | --- | --- | --- | --- | --- | --- |
| rs115517892 | 5 | 116050668 | 3720 | 0.68 | 0.27 | 0.04 | 0.01 | <0.01 |
| rs2898892 | 15 | 102115561 | 2506 | 0.48 | 0.47 | 0.02 | 0.02 | <0.01 |
| rs77855328 | 2 | 54521245 | 2892 | 0.66 | 0.15 | 0.14 | 0.03 | 0.01 |
| rs61623602 | 1 | 81931342 | 3151 | 0.67 | 0.26 | 0.05 | 0.02 | 0.01 |
| rs12675568 | 8 | 11814687 | 2608 | 0.31 | 0.55 | 0.05 | 0.08 | 0.01 |
| rs851028 | 6 | 35990153 | 2342 | 0.57 | 0.36 | 0.04 | 0.03 | <0.01 |
| rs78392151 | 17 | 10189354 | 3047 | 0.65 | 0.29 | 0.04 | 0.02 | <0.01 |
| rs34003417 | 8 | 143526909 | 3336 | 0.56 | 0.21 | 0.16 | 0.06 | 0.02 |
| rs4470854 | 6 | 96007436 | 2608 | 0.10 | 0.51 | 0.06 | 0.28 | 0.05 |
| rs146904513 | 9 | 8243307 | 3898 | 0.46 | 0.38 | 0.07 | 0.06 | 0.02 |
| rs8079676 | 17 | 64236953 | 3551 | 0.06 | 0.73 | 0.02 | 0.19 | <0.01 |
| rs77653134 | 10 | 25630272 | 3427 | 0.74 | 0.22 | 0.03 | 0.01 | <0.01 |
| rs138489075 | 13 | 108512103 | 3463 | 0.72 | 0.22 | 0.04 | 0.01 | <0.01 |
| rs61904837 | 11 | 93650581 | 2949 | 0.67 | 0.25 | 0.05 | 0.02 | <0.01 |
| rs35874160 | 1 | 4375288 | 3886 | 0.56 | 0.35 | 0.05 | 0.03 | 0.01 |
| rs33998921 | 2 | 76405567 | 3340 | 0.12 | 0.64 | 0.03 | 0.18 | 0.02 |
| rs453679 | 19 | 3300339 | 2211 | 0.57 | 0.40 | 0.01 | 0.01 | <0.01 |
